# The skin color and gender of high-fidelity simulation manikins in US simulation centers and their use in cultural humility training

**DOI:** 10.1186/s41077-026-00408-z

**Published:** 2026-01-21

**Authors:** Marie Anderson Wofford, Cortlyn Brown, Bernard Walston, Heidi Whiteside, Joseph Rigdon, Philip Turk

**Affiliations:** 1 Vice Director for Diversity, Equity, and Inclusion, Department of Emergency Medicine, Atrium Health Carolinas, Charlotte, NC USA; 2 University of North Carolina Chapel Hill School of Medicine, Chapel Hill, NC USA; 3 Department of Biostatistics and Data Science, Wake Forest University School of Medicine, Winston-Salem, NC USA; 4 Department of Data Science, University of Mississippi Medical Center, Jackson, MS USA

**Keywords:** High-fidelity simulation, Manikins, Cultural humility, Skin-tone, DEI

## Abstract

**Objective:**

Our objectives were to evaluate what proportion of simulation centers use high-fidelity simulation manikins to teach cultural humility, and to evaluate if manikin skin color and sex breakdown are representative of the USA population.

**Methods:**

Surveys were sent to simulation centers from our simple random sample. Key outcomes included skin color and gender of manikins and if cultural humility was taught via simulation. Point and interval estimates were calculated for the proportion of light-, medium-, and dark-colored manikins, the proportion of female and male manikins, and the proportion of centers using simulation to teach cultural humility. Confidence intervals were employed to test the null hypothesis that light/medium/dark skin color was 60/20/20 and female/male was 50/50 which is extrapolated from the US Census data.

**Results:**

Our response rate was 75% (41/55). All of the 41 responding simulation centers had manikins (1.0000, 95% CI: 0.9180, 1.0000). Twenty seven of the 41 simulation centers with manikins reported simulation scenarios with cultural humility (0.6585, 95% CI: 0.5127, 0.8044). Proportions of light-, medium-, and dark-colored manikins were 0.5150 (0.4669, 0.5625), 0.3086 (0.2651, 0.3547), and 0.1764 (0.1563, 0.1964), respectively. Proportions of male and female manikins were 0.6402 (0.6163, 0.6640) and 0.3598 (0.3360, 0.3837). The null hypotheses that skin color follows a 60/20/20 split and gender follows a 50/50 split were rejected.

**Conclusions:**

Most simulation centers surveyed teach cultural humility via simulation, however their manikins do not reflect the sex and skin tone of the USA population.

**Supplementary Information:**

The online version contains supplementary material available at 10.1186/s41077-026-00408-z.

## Introduction

Health inequities disproportionately affect racial and gender minorities in the United States, contributing to worse outcomes across a range of conditions and healthcare settings [1]. As population diversity continues to rise globally, including in the U.S. and Europe, health systems face increasing pressure to ensure that healthcare professionals are equipped to provide equitable, culturally responsive care [2, 3]. Cultural humility (CH) training—defined as a lifelong commitment to self-evaluation, redressing power imbalances, and fostering respectful partnerships—is one method of addressing these inequities [4]. Unlike cultural competence, which implies mastery of knowledge about other cultures, cultural humility emphasizes reflection, active listening, and awareness of implicit bias.

Simulation-based education has emerged as a powerful tool in health professions training, valued for its ability to replicate complex, real-world clinical encounters in a safe and controlled environment. High-fidelity simulation manikins (HFSM), in particular, are lifelike and responsive, capable of providing both verbal and non-verbal feedback, and have been shown to enhance learner performance compared to lower-fidelity modalities [5, 6]. However, the effectiveness of simulation is grounded in realism.

While simulation is a widely accepted modality of mimicking real-world scenarios, there is very little known about the skin tone and sex breakdown of simulation manikins across the USA. Foronda et al. studied simulation catalogs at an international conference and concluded that 94% of simulation body parts and manikins were white while only 4% were black [7]. While Foronda et al. showed a lack of diverse simulation manikins, a pilot study by Paroz et al. utilized simulated patients to teach cultural competency, and their innovative study showed improvement in learners’ comfort levels in treating patients from different cultural backgrounds [8]. 

In addition, there is a scarcity of published literature assessing if manikins at simulation centers are used to teach CH, as noted by an integrative review by Foronda et al.[9] They reviewed simulation learning themes and found that while cultural sensitivity and competence, insight and understanding, communication, and confidence and comfort were mentioned, there were no studies that mentioned simulation and CH.

Our study had two objectives: (1) to assess whether the sex and skin color distribution of HFSM in U.S. simulation centers aligns with U.S. Census demographics, and (2) to determine what proportion of centers use simulation to teach cultural humility. We hypothesized that the manikins would not reflect the racial and gender diversity of the U.S. population and that many centers would not yet be using HFS to teach CH.

Addressing these questions is critical not only for U.S. educators but for global stakeholders in simulation-based education, as implicit bias and representational gaps are not country-specific problems. These findings may inform broader efforts to enhance equity and realism in simulation design and practice worldwide.

## Methods

The study was deemed exempt by the Atrium Health Carolinas Institutional Review Board and was designed to comply with quality standards for survey reporting in medical literature [10]. 

### Survey design

As there were no questionnaires that addressed the study objectives, we worked with an expert survey methodologist on the creation of the survey which was then piloted to our intended audience. Through this process, several improvements were made. These included changing the skin color from ethnicity-defined (e.g., African American) to color tone-defined (light, medium, and dark) and the oversimplification of the gender options to male vs. female to reflect purchasing options. Response burden was decreased through skip patterns and breaks. The final questionnaire (Supplemental Digital Appendix 1) consisted of 10 questions grouped into (1) demographics, (2) skin tone and sex breakdown of HFSM, and (3) utilization of HFSM to teach CH. Participants generally completed the questionnaire within five minutes. The questionnaire did not include any identifying information requiring blinding.

In the original survey, the two questions in Table 1 had the following answer choices: it costs too much to purchase multiple manikins, did not know there were different skin colors/genders available, did not think that having different skin colors/genders mattered, and “other”. The “other” answer choice in the survey was a free response. However, as we reviewed the free responses, we found common themes and categorized them as shown in Table 1.

**Table 1:** Qualitative analysis of themes for why HFS manikins do not match the USA population in terms of skin tone and sex

	Respondents (*n* = 41)
If you noticed that you have very few high-fidelity simulation manikins with one skin color, why do you believe that is the case?	
Costs too much	8 (20%)
Did not know there were different skin colors available	3 (7%)
Older manikins (not available at time of purchase)	13 (32%)
Other^1^	13 (32%)
Missing^2^	4 (10%)
If you noticed that you have very few high-fidelity simulation manikins from one sex, why do you believe that is the case?	
Costs too much	7 (17%)
Did not know there were different sexes available	2 (5%)
Did not think that having different sexes mattered	1 (2%)
Genitalia is interchangeable	10 (24%)
Female manikins (not available at time of purchase)	3 (7%)
Other^1^	8 (20%)
Missing^2^	10 (24%)

^1^ Free response that did not fit other themes

^2^ No response

### Survey administration

We distributed our survey to directors of simulation centers across the USA from April 2021 through September 2021 via email with one follow-up email and two subsequent follow-up phone calls for non-respondents.

### Statistical analysis

For our primary objective, we drew a simple random sample without replacement of size 55 from the population of all 559 simulation centers in the country. Because the target population of interest in our case is finite in size, care must be taken in using standard statistical inference methods which assume sampling from an infinite size population. As a rule of thumb, the statistical underpinning for many basic methods apply reasonably well for samples taken without replacement provided the sample size is no larger than 10% of the population size [11]. Hence, we chose a sample size of roughly 10% of the population size of all 559 simulation centers in the country to address the study objective.

To have an idea of the precision associated with our estimation procedure in the null case, we ran an initial simulation study using a program written using the statistical software program R. For a sample of size 55 simulation centers (approximately 10% of the population size), we simulated draws from a multinomial distribution with probabilities 0.60, 0.20, and 0.20. Given limited prior information, the number of manikins for each simulation center was determined by taking a random draw from a discrete uniform distribution on 1-to-13 manikins. We than generated 1,000 resamples (by simulation center) to obtain a set of 98.3% bootstrap percentile confidence intervals. The margin of error for all three confidence intervals was roughly 0.05 for all skin colors with 95% familywise confidence. Similarly, using the same approach for gender, the margin of error for the two confidence intervals was roughly 0.05 for both genders with 95% familywise confidence. It was felt that this level of precision was more than adequate for estimation of these proportions. For estimating the proportion of all simulations centers that have HFSM, exact confidence limits were obtained based on the hypergeometric distribution.

The primary study objective was to estimate the proportion compositions of skin color and sex of the HFSM used by all simulation centers across the country to see if they reflect the USA population. As we were unable to identify accurate data on the skin tone variations in the USA population, we extrapolated skin tone from the 2020 USA Census race and ethnicity data [12]. The proportion of “White alone (not Hispanic)” were classified as light-skin, and the proportion of people who identified “Asian alone,” “American Indian,” “Pacific Islander,” “Two or more races,” “Hispanic or Latino,” and “Black alone” were split into approximately even proportioned groups of medium- and dark-skinned manikins (20% of the population each).

As manikin skin color (light, medium, dark) and sex (male, female) are multinomial data clustered by simulation center, the data are likely correlated [12, 13]. To estimate the proportions of interest, we fit a generalized linear mixed model with an intercept as a fixed effect and simulation center random effects. We conducted likelihood ratio tests on the simulation center random effects variance component(s) to test for significant center-to-center variability with respect to the multinomial data. We then marginalized and refit the model and inverted the link function to recover estimates of the proportions. Stratified block bootstrapping was used to obtain a set of bootstrap percentile confidence intervals where resampling was done by simulation center to preserve the correlation structure among the data [14]. The confidence level was chosen to preserve familywise alpha at 0.05. Hypotheses about representation (60/20/20 light/medium/dark and 50/50 male/female) were tested by examining whether or not the null hypothesized values were all contained by the bootstrap confidence intervals.

Because it cannot be assumed with certainty that every simulation center will have HFSM, we initially estimated the proportion of all simulation centers that have them along with a 95% confidence interval. The second study objective was to estimate the proportion of centers that used HFS to teach CH. The survey was written such that this was a yes/no question to be answered at the center level. Consequently, we used the sample proportion and associated 95% confidence interval to estimate the proportion of centers that used HFS to teach CH. Statistical analyses were performed using R version 4.1.2.

## Results

Of the 55 simulation centers in the sample, 41 responded (75% response rate). One of the simulation centers reported slightly different total numbers of HFSM for color and sex. Hence, the total number of reported high-fidelity simulation manikins for this sample was 499 for color and 503 for sex. The average number of reported HFSM for this sample was roughly 12, with a range of 3-to-48 HFSM.

**Fig. 1:**
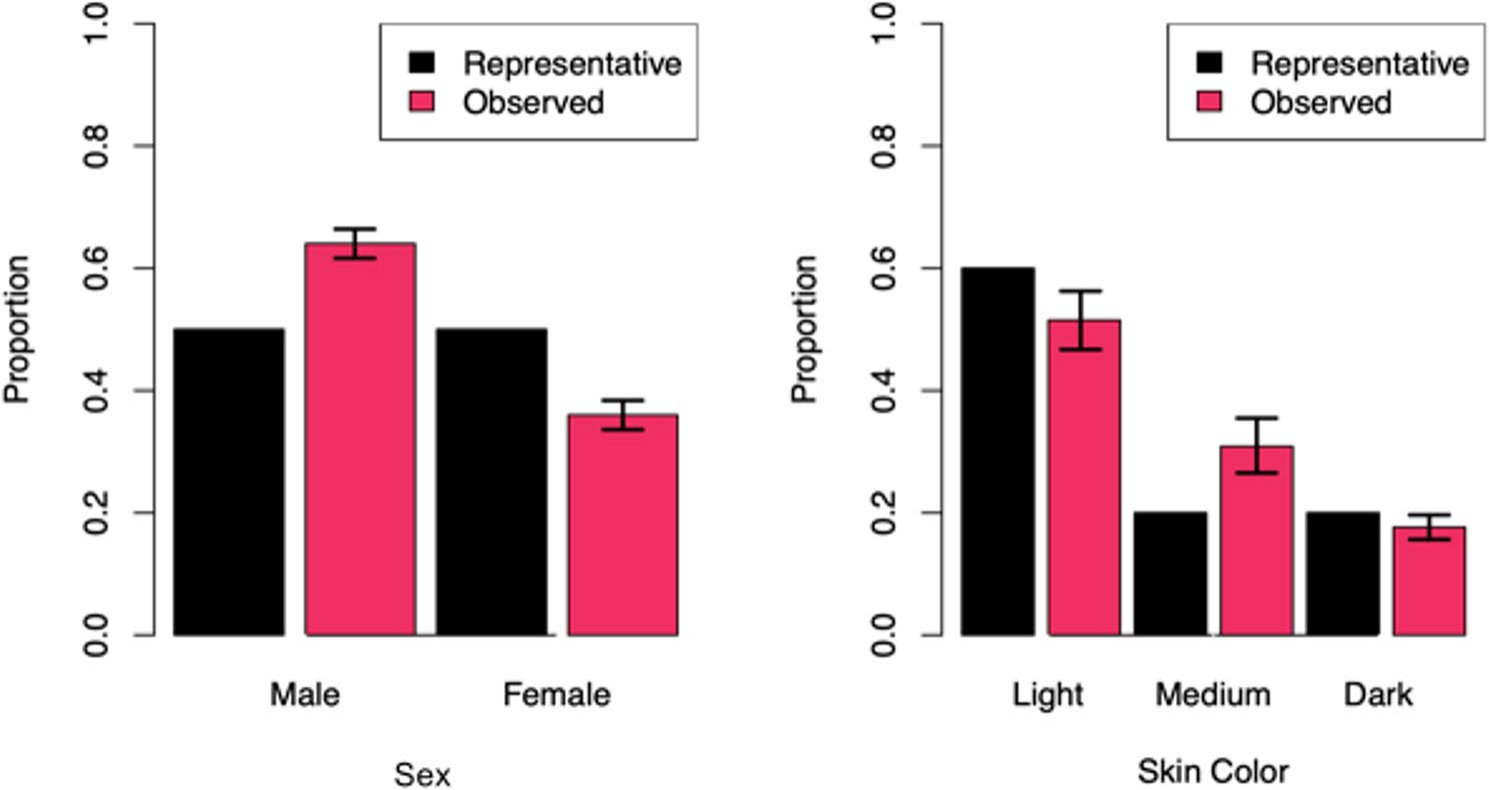
Representativeness of HFSM sex and skin color. Error bars represent 95% confidence intervals

Point estimates of the skin color proportions for HFSM from simulation centers across the country are 0.5150, 0.3086, and 0.1764 for light, medium, and dark skin colors, respectively. Simultaneous bootstrap percentile confidence intervals for these proportions (familywise confidence level 95%) using resampling by simulation center were [0.4669, 0.5625], [0.2651, 0.3547], and [0.1563, 0.1964] for light, medium, and dark skin colors, respectively (Fig. 1). As none of the confidence intervals contain their corresponding null values of 0.60, 0.20, and 0.20, that the proportion breakdown of skin color of the HFSM used by all simulation centers across the country does not reflect the USA population. Similarly, point estimates of the sex proportions for HFSM from simulation centers across the country are 0.6402 and 0.3598 for male and female manikins, respectively. Simultaneous bootstrap percentile confidence intervals for these proportions (familywise confidence level 95%) using resampling by simulation center were [0.6163, 0.6640] and [0.3360, 0.3837] for male and female manikins, respectively (Fig. 1). As neither confidence interval contains its null value of 0.50, the proportion breakdown of sex of the HFSM used by all simulation centers across the country does not reflect the USA population.

We also fit a generalized linear mixed model to these multinomial data with an overall intercept as a fixed effect and simulation center random effects and ran likelihood ratio tests on the simulation center random effects variance component(s). There was strong evidence to suggest significant center-to-center variability for both skin color (p-val < 0.0001) and sex (p-val = 0.0019).

All of the 41 responding simulation centers had manikins (1.0000, 95% CI: 0.9180, 1.0000). For objective two, 27/41 (0.6585, 95% CI: 0.5127, 0.8044) of centers responded “yes” to using HFS to teach CH. Of those 27 programs, 24 responded with the percentage (0–100) of total HFS cases using CH as a primary or secondary learning objective, and the mean response was 23.8 (SE: 4.6) (Table 2). The most common reasons for lack of skin color diversity were older manikins and cost. The most common reasons for lack of sex diversity were interchangeability and cost (Table 1).

**Table 2:** Quantitative analysis of high-fidelity simulation manikins used by simulation centers; skin and sex diversity and their use in CH training

	Total Manikins
Manikin gender (*n* = 503)	
Male	322 (64%)
Female	181 (36%)
Manikin skin color (*n* = 499)	
Light	257 (52%)
Medium	154 (31%)
Dark	88 (18%)
Cultural humility as a learning objective for HFS cases (*n* = 41)	
Yes	27 (66%)
No	14 (34%)
Percent of HFS cases with cultural humility as a primary or secondary learning objective (mean ± standard error among *n* = 24 responders)	23.8 (SE: ± 4.6)

## Discussion

In this study, we assessed the skin tone and sex diversity amongst HFSM at simulation centers across the United States as well as if these simulation manikins were used to teach CH. Our data suggests that while most simulation centers use HFS to provide CH training, the HFSM do not reflect the skin tone and sex of the USA population.

While there are many possible reasons for this skin tone and sex disparity, a common theme among our survey respondents was that they were not aware that different skin color options or sexes were available. Literature suggests that simulation manikin developers are advertising far more lighter skin tone than darker skin tone manikins and body parts, and this possibly contributes to the disparity in diversity [15]. In addition, simulation manikins are costly at $19-25k regardless of skin tone. Therefore, simulation centers do not frequently purchase new manikins. It is possible that when many of the simulation manikins used at simulation centers were purchased, there were limited options for female sex or darker skin manikins. In particular, 32% of respondents reported that one of the reasons for lack of diversity within skin color was the limited availability of skin color options when they purchased their manikins. Survey respondent comments speak such as “many of our [manikins] are older and there was not an option when purchased [for different skin tones or female sex]” speak to this. For example, Laerdal Medical did not release the Nursing Anne simulator in medium and dark skin tones until 2018. Laerdal also released an African descent upgrade which allows the interchanging of faces to medium and dark skin tones, but this was not done until 2020. The release of these options coincided with increased institutional and public attention to structural racism in healthcare, particularly following the global Black Lives Matter Movement. This period marked a broader push within medical education to improve diversity, equity, and inclusion (DEI), leading to greater scrutiny of representational practices in educational tools [16, 17]. Other survey respondents, however, provided alternative explanations including “manikins purchased prior to greater awareness of racial disparities” or that they “never ‘needed’ a female [manikin]” with another center saying, “the female simulators we have are great for maternal simulations, but the male [manikins] work better for most of our scenarios.” Another stated that “some colors have proven to be easier to keep clean.” Many of these themes mentioned were also reported by Foronda et al..[18] Their study, however, did not focus on HFS.

We believe it is essential for high-fidelity simulation manikins (HFSM) to reflect the diversity of the U.S. population—and by extension, the patient population that trainees will encounter—for several important reasons. Simulation-based education relies on realism as one of its core pillars. However, certain modifications currently used to represent diverse patients can actually detract from that realism. For example, we have observed instances where a pink shirt is placed on a phenotypically male manikin to signify a female patient, or where an afro wig is placed on a light-skinned manikin to signify a Black patient. Several centers reported using wigs or interchangeable genitalia to simulate different genders. One respondent stated, “We are able to interchange genitalia on all mannequins to simulate other gender. We add wigs as well when running female scenario.” Yet another noted that “even manikins designed to be convertible between genders still appear overwhelmingly masculine.” These modifications also have the potential to increase sex and racial biases of the learners. In addition, if learners are only exposed to light-skin male manikins, that could potentially perpetuate the concept of light-skin being the standard while everything else being “other.” Using the socially dominant demographic of light-toned male manikins in simulation to teach CH may be damaging to the message that is trying to be relayed through CH training.

We recommend the creation of institutional and national grants aimed at providing the financial means so that centers can purchase new and diverse HFSM. Although having an increased budget to purchase new diverse manikins is ideal, there are several more cost-effective alternatives such as the us1e of realistic Mask-Ed™ masks. Crownover et al. conducted a study where a faculty member wore a mask resembling an older adult while adjusting gait and other mannerisms [19]. The group of nursing students that were participants in the study were able to learn more about the care of older adults while addressing misconceptions, stereotypes, and biases they may have when caring for older adults [20]. While this study focuses on live simulation instead of manikins, it shows how making simulation more realistic enhances how learners are engaged and how simulation improves care for specific populations. A last option to increase the diversity of an individual center’s HFSM is to collaborate with local centers and professional society chapters and create local sharing networks of simulation manikins.

It is also important to have simulation scenarios focused on CH. There are several ways to accomplish this. Roberts et al. suggests modifying the biologic aspect of the scenario, such as the pulse, blood pressure, and respiratory rate in response to cross-cultural interactions (such as a change in heart rate or an avoidant eye gaze when a male provider describes a sensitive exam to a female Muslim patient) [20]. In addition, Roberts et al. recommends utilizing all aspects of the setup such as language (having the HFSM respond in a non-English language). Not only would it allow the trainee to gain comfort in interacting with patients with limited English proficiency, but it could also teach basics of interpreter usage such as positioning of the interpreter in relation to the provider and patient. While it is possible to create your own scenarios for simulation, the Organization of Associate Degree Nursing and Unbound Medicine offers an online webinar on incorporating Diversity, Equity and Inclusion curricula into simulation [21]. This webinar, however, is geared towards the nursing field and does not focus on HFSM. We, therefore, advocate for the creation of a free-access database of HFS ready-made multidisciplinary scenarios focused on CH.

In addition to increasing the diversity of HFSM and their usage to teach CH at an institutional level, we recommend that manufacturers increase the diversity of their product offerings, focusing on creating phenotypically true representations of different genders and ethnicities rather than just “merely changing the pigment of the skin” as one of our respondents noted. It is also important for simulation manikin companies to advertise through a lens of equity and for national simulation groups to lead the charge through position statements and calls to action.

**Fig. 2:**
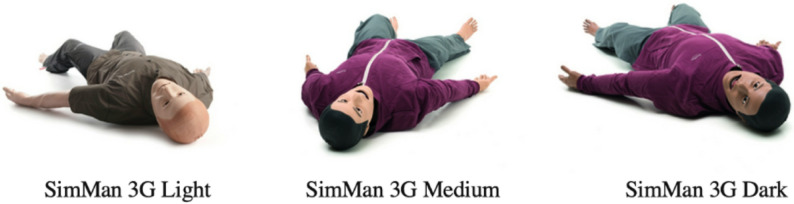
Laerdal SimMan 3G in light, medium, and dark skin-tone

There are several limitations of our study. First, we acknowledge a key limitation in the way we categorized racial and ethnic groups into skin tone classifications (light, medium, dark) to align with the limited options provided by high-fidelity simulation manikin manufacturers. This framework is inherently reductive and does not capture the broad phenotypic diversity that exists within any racial or ethnic group. However, in the absence of standardized definitions or more detailed manufacturer data, we made this decision to enable a meaningful comparison between available manikin skin tones and U.S. Census demographic data. We recognize that this simplification may reinforce inaccurate associations between race, ethnicity, and skin tone, and we emphasize the need for more nuanced and inclusive classification systems in future research and in simulation product development. An additional limitation is that several survey respondents stated that they use simulation manikins with replaceable genitalia to symbolize both male and female patients while saving on cost. To minimize any confusion this may cause, our survey instructed respondents to select the sex that most adequately represents the non-interchangeable phenotypic features of the manikin such as presence or absence of breast tissue, hip proportion, and facial structure etc. Lastly, the pictures in our survey that served as examples for light-, medium-, and dark-skin tone HFSM (Fig. 2) were taken from one company, and it is possible that each company has slightly different variations of light-, medium-, and dark-skin tone simulation manikins. We also want to acknowledge that gender is a more appropriate marker for diversity, but the study was reduced to binary sex identities to reflect the limited purchasing options for HFSM. Lastly, our study did not account for regional or neighborhood-level differences in racial or gender demographics. While we used national census data for comparison, we recognize that individual institutions may serve highly diverse or unique local populations. As such, future research should incorporate more localized demographic data to better evaluate how well simulation manikins reflect the communities they are intended to represent.

### Implications

This study serves as the first piece of literature on the skin tone and gender breakdown of HFSM utilized by simulation centers in the USA. While HFS is already being used by most simulation centers surveyed to teach CH, there are still large gaps in gender and skin tone diversity of HFSM. Future studies will focus on understanding how simulation centers use HFS to teach CH, the effectiveness of teaching CH via HFS, and any barriers to obtaining HFSM that reflect the USA population.

## Supplementary Information

Supplementary Material 1

## Data Availability

No datasets were generated or analysed during the current study.
