# Supplementary material for: The skin color and gender of high-fidelity simulation manikins in US simulation centers and their use in cultural humility training
Source: Adv Simul (Lond). 2026 Jan 21;11:9. doi: 10.1186/s41077-026-00408-z (PMC12908334; doi:10.1186/s41077-026-00408-z)
Supplement: Supplementary file 1 — Supplementary Material 1 [file 41077_2026_408_MOESM1_ESM.pdf]

Diversity of Simulation Mannequins and their use in Cultural Humility Training

Thank you for participating in our research study on the racial and gender diversity of high-fidelity simulation mannequins' and their use in cultural humility training.

The study is being conducted by the Department of Emergency Medicine at Atrium Health's Carolinas Medical Center and was deemed exempt by the Atrium Health Institutional Review Board. The principal investigator is Cortlyn Brown, MD.

Completion of the survey is completely optional and constitutes your consent to participate in this study. If at any time you want to stop your participation, you can without any repercussions.

If you have any questions during the completion of the survey, please pause the survey, and reach out to the study investigators via email.

All data collected in this study will be protected and will be stored electronically on secure servers in the United States of America in limited-access folders until the study is complete. Identifying variables will be deleted prior to data analysis. Data will never be reported in a way that identifies institutions or individuals.

Contact
If you have any questions regarding this study, please contact Cortlyn Brown, MD at cortlyn.brown@atriumhealth.org.

---

1 What is the name of your simulation center/institution?

*This will remain anonymous during data analysis.

____

---

2 Select the region your simulation center is in.

◯ Northeast: New England (ME, NH, VT, MA, RI, CT) ◯ Northeast: Mid Atlantic (NY, NJ, PA) ◯ Midwest: East North Central (OH, MI, IN, WI, IL) ◯ Midwest: West North Central (MN, IA, MO, ND, SD, NE, KS) ◯ South: Atlantic (DE, MD, WV, VA, NC, SC, GA, FL, DC) ◯ South: East South Central (KY, TN, AL, MS) ◯ South: West South Central (AR, LA, OK, TX) ◯ West: Mountain Division (MT, ID, WY, CO, NM, AZ, UT, Nevada) ◯ West: Pacific Division (CA, OR, WA, AK, HI)

---

3 What is the total number of high-fidelity simulation mannequins (from infants to adults) that you have in your simulation center (type number here)?

For study purposes: high-fidelity simulation mannequins refer to computerized full-bodied simulators. These simulators often have physiological responses (eg. sweating, seizure activity, and vomiting) and the ability to interact and communicate.

____

Light

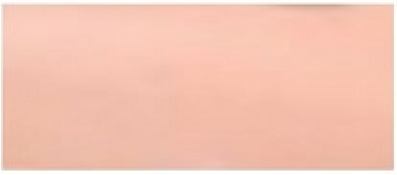

Medium

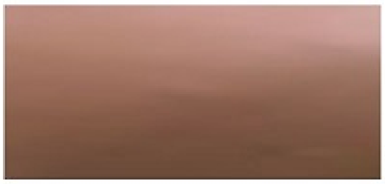

Dark

4 Write the total number of high-fidelity simulation mannequins (from infants to adults) that you have with each skin color (this should add up to the total number of high-fidelity simulation mannequins from question 3).

* required

Number of light color mannequins ______
Number of medium color mannequins ______
Number of dark color mannequins ______

5 If you noticed that you have very few high-fidelity simulation mannequins with one skin color, why do you believe that is the case (select the best answer)?

◯ A: It cost too much to purchase multiple mannequins ◯ B: Did not know there were different skin colors available ◯ C: Did not think that having different skin colors mattered ◯ D: Other

If other, explain ________________________________

6 How many high-fidelity mannequins (from infants to adults) do you have from each gender? These should add up to the total number of high-fidelity simulation mannequins from question 3.

Although you can change the genitalia on many simulation mannequins, answer based on the overall appearance of the mannequin.

* required

Number of female mannequins ______
Number of male mannequins ______

7 If you noticed that you have very few high-fidelity simulation mannequins from one gender, why do you believe that is the case (select the best answer)?

◯ A: It cost too much to purchase multiple mannequins ◯ B: Did not know there were different genders available ◯ C: Did not think that having different genders mattered ◯ D: Other

if other, explain

____

8 Cultural humility is defined as a practice that promotes personal reflection and growth around culture with the goal of increasing the provider's awareness.

Do learners at your simulation center (RNs, MDs, DOs, RTs, etc.) participate in high-fidelity simulation scenarios where an aspect of cultural humility is one of the primary or secondary learning objectives (eg, a resident navigating a trauma scenario where a Jehovah's witness is actively bleeding and has a hemoglobin of 5)?

◯ Yes ◯ No

What percent of high-fidelity simulation scenarios that your learners encounter have an aspect of cultural humility as one of the primary or secondary learning objectives?

____

For cases where an aspect of cultural humility is a primary or secondary learning objective, please mark any modifications you make to the high-fidelity mannequin (select all that apply).

☐ Put on wig/facial hair to represent a specific ethnicity ☐ Put on clothing to represent a specific ethnicity
☐ Have the mannequin have an accent or use specific vernacular to represent a specific ethnicity
☐ Put on wig/facial hair to represent a specific gender ☐ Put on clothing to represent a specific gender
☐ Actively select a narrator for the mannequin to represent the gender of the mannequin
☐ Other

If other modification, explain

____

6. Use the space below to provide any additional thoughts.
